# Genetic Variation in the* IL-6* and* HLA-DQB1* Genes Is Associated with Spontaneous Clearance of Hepatitis C Virus Infection

**DOI:** 10.1155/2016/6530436

**Published:** 2016-06-02

**Authors:** Paul Ravi Waldron, Ilana Belitskaya-Lévy, Aarthi Chary, Johann Won, Mark Winters, Alexander Monto, James Ryan, Laura C. Lazzeroni, Mark Holodniy

**Affiliations:** ^1^VA Palo Alto Health Care System, Palo Alto, CA 94304, USA; ^2^Division of Infectious Diseases & Geographic Medicine, Stanford University, Stanford, CA 94305, USA; ^3^Cooperative Studies Program Coordinating Center, VA Palo Alto Health Care System, Palo Alto, CA 94304, USA; ^4^San Francisco VA Medical Center, San Francisco, CA 94121, USA; ^5^University of California, San Francisco, San Francisco, CA 94143, USA; ^6^Department of Psychiatry, Stanford University, Stanford, CA 94305, USA

## Abstract

*Background*. Millions of people are infected with hepatitis C virus (HCV) worldwide and 30% spontaneously clear the infection. Reasons for HCV clearance without antiviral treatment are not well understood.* Methods*. Blood was collected for DNA analysis from patients with chronic HCV infection or evidence of spontaneous clearance. To overcome anticipated limitations of small sample size, primary analyses consisted of a candidate gene analysis of 12 preselected genes based on known association with host immunologic response to HCV infection. To further reduce the impact of multiple testing on power, a single likelihood ratio test was conducted for each gene using all associated SNPs assayed on the Illumina Quad 610/660W chip. Step-down permutation methods were used to adjust for multiple testing in all analyses.* Results*. Ninety-five and 62 patients with HCV chronic infection or spontaneous clearance, respectively, were included for analysis.* HLA-DQB1* (*p* = 1.76⁎10^−5^) and* IL-6* (*p* = 0.0007) genes were significantly associated with spontaneous HCV clearance.* IL-28B* was not significantly associated with spontaneous clearance (*p* = 0.17).* Conclusion*. Our whole-gene analytic strategy identified a previously unreported association of IL-6 with spontaneous clearance of HCV infection. We also confirmed the finding that HLA-DQB1 is associated with spontaneous resolution of HCV infection.

## 1. Introduction

The hepatitis C virus (HCV) is a flavivirus which primarily affects the liver, with a seroprevalence of over 185 million infections worldwide [1]. Approximately 70% of people infected with HCV develop chronic infection and 30% are known to spontaneously clear the infection; however these rates vary by ancestry, gender, and other factors [2–4]. Chronic infection with HCV results in hepatic inflammation, necrofibrosis, and may ultimately cause cirrhosis, hepatocellular carcinoma, and death due to liver failure [3]. Factors that lead to spontaneous clearance may provide important insight into disease pathogenesis and may be important for future assessments of prognosis as well as guiding discovery of novel therapies.

It has been shown that mutations that alter the expression of certain antiviral cytokines are predictive of HCV treatment response and disease prognosis and therefore may also be instrumental in the biology of spontaneous clearance. Single-nucleotide polymorphisms (SNPs) rs12979860 and rs8099917, which are upstream of* interleukin 28B* (*IL-28B*, also known as interferon-*λ*3) and in strong linkage disequilibrium (LD) with each other, have been implicated as predictors of treatment response in separate studies [5–7]. These findings were confirmed with candidate gene studies and genotyping currently can be used clinically to advise patients on the likelihood of success prior to interferon-based HCV treatment [5].

Polymorphisms may also affect cell-mediated immune responses in part due to their effect on viral antigen presentation via HLA class II molecules. A specific* HLA* class II allele *DQB* ^*∗*^0301 was found by de Rueda et al. in a candidate gene analysis of samples from 428 treated HCV infected patients to correlate significantly with favorable treatment response via candidate gene analysis [8]. This suggests that* HLA-DQB1* is important for presentation of HCV antigens and resulting clearance of virus.

A recent genome-wide association study (GWAS) on a multicohort sample of 919 HCV spontaneous clearers and 1,482 patients with persistent infection identified genome-wide significant associations with rs12979860 in the promoter region of* IL-28B* and rs4272729 adjacent to *DQB* ^*∗*^0301 [9]. Together these SNPs explained 15% of the variation in HCV resolution in a sample comprising patients with European and African ancestry [9]. While GWAS provides an exhaustive analysis of the association of individual SNPs with a clinical outcome, it requires a large number of patients to empower these studies and minimize type I error [8, 9]. In addition, GWAS may not identify SNPs that may not reach significance individually but may be found to be important in aggregate. Whole-gene analysis is a technique for detecting the aggregate association of SNPs on a given gene with a clinical outcome that addresses these limitations of GWAS. Herein, we describe our whole-gene analysis for selected genes that are likely to be associated with HCV clearance.

## 2. Subjects and Methods

### 2.1. Ethics Statement

The study was approved by both the Stanford University and the University of California at San Francisco Institutional Review Boards and was conducted in accordance with the guidelines established by the Declaration of Helsinki. Written informed consent was obtained from all patients.

### 2.2. Subject Recruitment and Enrollment

One hundred and sixty patients were recruited from 3 sites in the San Francisco Bay Area. Of these, 122 patients were enrolled at both the San Francisco Veterans Affairs Medical Center and the University of California at San Francisco Moffitt-Long Hospital from July 2007 to December 2009 as part of a study of immune responses related to hepatitis C. Patients included in the study were those with confirmed positive HCV antibody and sufficient DNA in extracted samples over six months, although all patients had infection status followed over many years. Another 38 patients were enrolled at the Veteran's Administration Palo Alto Health Care System between July 2007 and September 2011 as part of a cohort enrolled for the purpose of studying HCV infected host transcriptional responses to interferon as well as the host genomics of viral clearance. Blood was drawn at baseline and at 12, 24, 36, and 48 weeks after enrollment; patients from whom sufficient DNA was not obtained were contacted if possible and blood was redrawn.

### 2.3. Study Assays and Sample Preparation

Spontaneous clearance was defined as patients who were HCV antibody positive by screening EIA assay and confirmed by CHIRON® RIBA® HCV 3.0 SIA (RIBA) assay, who had a negative HCV viral load (Abbott Real time HCV assay, Abbott Diagnostics, North Chicago, IL) in plasma without a history of HCV-specific treatment or who had documented acute HCV infection by antibody and/or HCV viral load testing, and whose HCV viral load became undetectable without need for HCV-specific treatment. Chronic HCV infection was defined as those patients with a positive HCV antibody and positive HCV viral load result. HCV genotype was determined using the Versant INNO-LiPA HCV II line probe assay (Siemens Diagnostics, Tarrytown, NY) according to the manufacturer's recommendations. HIV-1 antibody testing was performed using a screening HIV1/HIV2 EIA and confirmatory western blot assay. This study was approved by the Stanford University and the University of California at San Francisco Institutional Review Boards.

Blood samples were collected in cell preparation tubes (CPT) (Becton Dickinson, Franklin Lakes, NJ) and peripheral blood mononuclear cells (PBMC) were separated from whole blood according to the manufacturer's instructions, washed with PBS twice, and stored in freezing medium (10% dimethyl sulfoxide (DMSO)/90% fetal bovine serum (FBS)) or as dry pellets at −80°C. Cell DNA was purified from PBMC using the Qiagen kit systems (Qiagen, Valencia, CA). DNA purity was checked and any DNA samples with a 260/280 ratio of <1.7 were repurified. DNA amounts were standardized with PicoGreen.

Genotyping was performed using the Illumina Human610-Quad array (samples analyzed prior to 2011) or the Illumina Human660W-Quad array (Illumina, San Diego, CA) on an Illumina BeadStation system, with GoldenGate Genotyping and Infinium Whole-Genome Amplification procedures. Only SNPs evaluated on both chips were included in the final analysis. Each array had several sample-dependent and sample-independent internal controls which were monitored with the BeadStudio Genotyping Module Integrated Controls Dashboard. Initial analyses of data and data quality were accomplished with the BeadStudio software.

### 2.4. Candidate Gene Selection

Due to sample size limitations, we carried out a primary analysis of candidate genes. We performed a detailed literature analysis and identified 67 host genes known or hypothesized to be integral both to the host immune response and to the life cycle of HCV. This included genes involved in cell-to-cell signaling, both innate and adaptive immune responses including interferon effector genes involved with the Janus Kinase-Signal Transducer and Activator of Transcription (JAK-STAT) pathway, major histocompatibility complex mediated antigen presentation, and facilitation of HCV entry into cells. The initial list was further reduced to twelve candidates that were determined to have the most evidence supporting their role in HCV clearance, either spontaneous or treatment-related (Table 1). A single overall test of each candidate gene was based on all available SNPs either within the coding region of the gene of interest or within the region of the next single 3′ or 5′ flanking/adjacent gene included on the array as defined by the array manufacturer (Illumina) based on the available literature at the time of production.

Rs12979860 was identified in the literature as a SNP of special interest. However, it was only assayed on the Illumina Human610-Quad array. Therefore, for the 32 samples assayed with the Illumina Human660W-Quad array, a separate allelic discrimination RT-PCR assay was performed for rs129798690 as described by Tillmann et al., with results included in our analysis [10].

### 2.5. Statistical Analysis

Primary analyses focused on whole-gene tests of 12 preselected genes of interest as described above to reduce the impact of required multiple testing corrections on power, given the small sample. A genome-wide SNP-based analysis was also performed in the event that single SNPs in genes or regions not previously associated with HCV spontaneous clearance might be detected and serve to inform other candidate whole-gene analyses.

### 2.6. Data Preprocessing

Approximately 610–660 K SNP markers were genotyped in a genome-wide scan. SNPs were excluded from the analysis if they had a call rate of <0.85, a minor allele frequency <0.01, or a Hardy-Weinberg equilibrium *p* value < 0.0001, leaving 150 SNPs in the 12 candidate genes and 540,031 additional genome-wide markers (excluding sex chromosomes), which were designated as potential null SNPs to be used to control for population stratification. We used the following “100, 5, 0.5” criterion for LD pruning to derive null SNPs as follows: (1) consider a window of 100 SNPs; (2) calculate LD between pairs of SNPs in the window; (3) remove one of the pairs of SNPs if the LD is greater than 0.5; and (4) shift the window 5 SNPs forward and repeat the procedure. After linkage disequilibrium pruning, 309,470 null SNPs were identified. These data preprocessing steps were performed using SNP & Variation Suite version 8 (Golden Helix, Bozeman, MT) and PLINK [11].

### 2.7. Population Stratification

To control for population stratification, principal components analysis was used to derive “ancestry eigenvectors” from the set of 309,470 null SNPs. As self-identified race is not a perfect predictor of ancestry group, we used genotype-based principal components to adjust for population stratification. The genomic inflation factor, calculated before and after correction for population structure, was used to evaluate how successful the principal components were in correcting for population structure effects [12].

The eigenvectors were calculated using the Bioconductor R package* snpStats* and the R package* eigen* [13–15]. Significant ancestry eigenvectors were added as covariates in the logistic regression model of HCV clearance.

### 2.8. Gene-Based Analysis

An additive genetic model was used in all analyses. For each of the 12 selected genes, a single likelihood ratio test (LRT) compared two logistic regression models of HCV clearance: the “full” model with all SNPs for that gene as well as the ancestry eigenvectors included as predictor variables and the “null” model which included the ancestry eigenvectors as the only predictors. The whole-gene LRT produced a single* p* value for each gene, representing the total collective association of the SNPs in that gene with HCV clearance. Whole-gene tests have the potential to increase power by combining statistical signal across SNPs, while reducing the number of tests for which a multiple testing correction is needed. Many alternative gene-based methods of association have been proposed, especially for rare variant data [16]. The LRT method was selected to avoid complex or adaptive models that might require a larger sample for robust inference.

### 2.9. Step-Down Permutation Procedure to Adjust for Multiple Comparisons

To account for multiple testing of 12 separate candidate genes, a step-down permutation procedure was implemented as follows [17]. The clinical variables of the 157 patients (i.e., the outcome variable, covariates, such as HIV status, and the ancestry eigenvector) were permuted randomly with respect to their genotype data. The LRT tests above were repeated using the permuted data yielding 12 permutation LRT* p* values. These* p* values were ordered from smallest to largest and the permutation procedure was repeated 10,000 times to produce a permutation distribution of the smallest* p* values, the second smallest* p* values, and so on. The fifth percentiles of these distributions provided significance thresholds that were used to assess the significance of the 12 genes based on their observed LRT* p* values. The step-down procedure was applied as follows: if a gene with the smallest* p* value passed its corresponding significance threshold, it was declared significant and the next gene (with the second smallest* p* value) was evaluated. If the second gene passed its significance threshold, then it was declared significant and the next gene was evaluated, and so on. Once any gene did not pass its threshold, it and all the remaining genes were declared to be nonsignificant. The permutation step-down process provides strong control of familywise error rate (i.e., it ensures that the probability of more than zero false positives is 5% or less). Both stratified and random permutations were used and the results were compared. Stratified permutations were performed within the ethnic strata defined by the genotype-based principal components.

### 2.10. Univariate Analysis of Candidate SNPs

Each of the 150 SNPs in the candidate genes was also analyzed in a single-SNP logistic regression model adjusted for population stratification. To control for multiple comparisons, the step-down permutation procedure as the one described above was used.

### 2.11. Genome-Wide Scan

To perform a genome-wide association analysis of SNPs with HCV clearance, a single-SNP logistic regression was performed with HCV clearance as the outcome variable, with and without adjustment for the population genetic structure. Benjamini and Hochberg procedure was used to adjust the raw* p* values for multiple comparisons [18].

Most analyses were performed in R [13].* SNP_tools* [19] and* Haploview* [20] were used to create LD maps. D′ was used as the LD measure [21].

## 3. Results

Of the 163 patients initially recruited, three patients (1 spontaneous resolution and 2 HCV infected) were excluded from analysis due to insufficient DNA isolated. Three patients (1 spontaneous resolution and 2 HCV infected) were excluded from analysis due to gender mismatch. Demographic and clinical characteristics of the 157 study participants for which data could be obtained are presented in Table 2. Ninety-five patients (61%) had chronic infection and 62 (39%) had spontaneous resolution. Between the groups, only self-identified race was significantly different with African-American patients representing 13% of the spontaneous resolution group and 37% of the chronic infection group (*p* = 0.01).

### 3.1. Population Stratification

**Figure 1:**
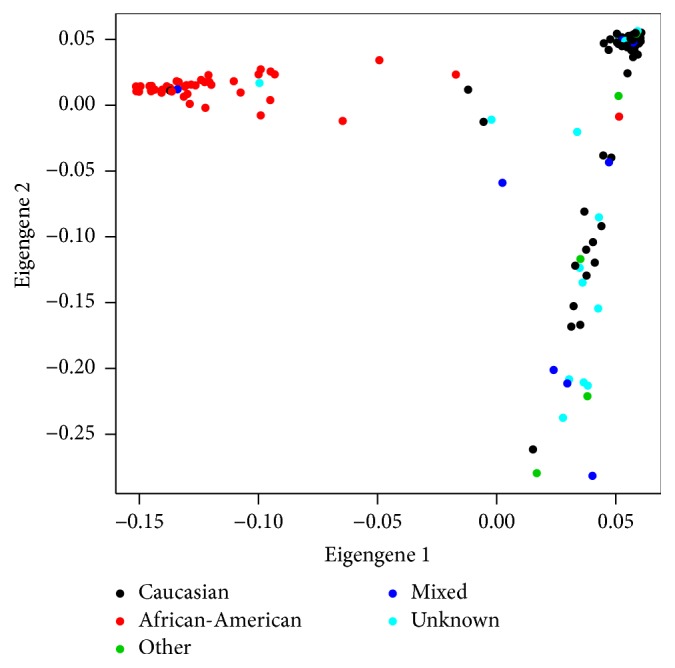
Population stratification via null genes.

Shown in Figure 1 is a two-dimensional representation of data on the first two principal components (“ancestry eigenvectors”). The first eigenvector provides a near perfect separation of Caucasian and African-American populations, the two largest groups in the study. When the first four principal components were entered in the logistic regression model of chronic infection versus spontaneous resolution, the first eigenvector was found to be significant (*p* = 0.0026). The genomic inflation factor was estimated to be 1.64 before correction for population genetic structure and 1.05 after correction using the first ancestry eigenvector. The quantile-quantile plots of the null SNP test statistics before and after correction for population stratification are provided in Supplementary Figure  1 in Supplementary Material available online at http://dx.doi.org/10.1155/2016/6530436. The first eigenvector was used in all subsequent analyses to adjust for population stratification. Using additional eigenvectors did not alter the results.

### 3.2. Gene-Based Analysis

Twelve candidate genes, including 150 SNP, were selected for primary analysis (Table 1).

The number of SNPs per gene ranged from 1 to 42. The left panel of Table 3 presents the observed LRT* p* value for each gene and the corresponding significance threshold used to adjust for multiple testing. The right panel of Table 3 presents results for a parallel analysis with HIV status included as a covariate. The same two genes,* HLA-DQB1* and* IL-6*, reached statistical significance in both analyses. The same results were obtained when stratified permutations were performed where strata, defined by the first ancestry eigenvector, roughly corresponded to Caucasian and African-American populations.

**Figure 2:**
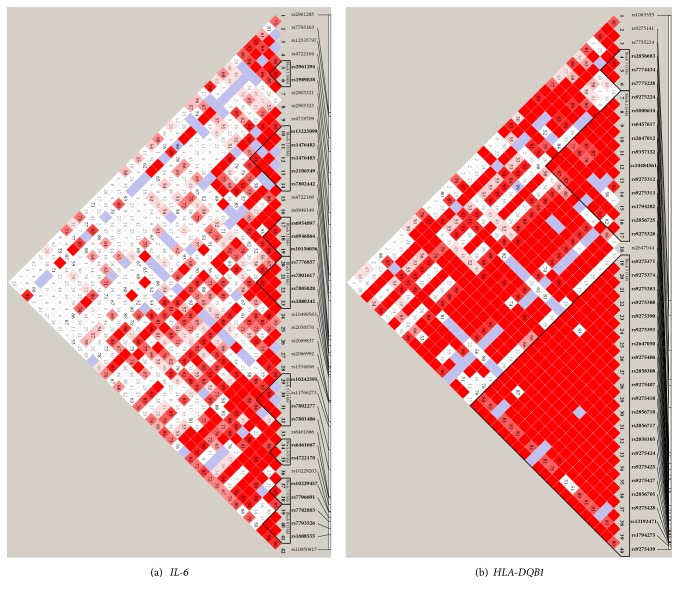
Linkage disequilibrium maps of the two significant genes, measured as D′.

Genotype counts and SNP frequencies for* HLA-DQB1* and* IL-6* are provided in Supplementary Table  1. LD maps for the two significant genes are presented in Figure 2 and for the remaining genes in Supplementary Figure  2. The LD map for HLA-DQB1 demonstrates a high degree of disequilibrium, which may indicate that a few SNPs in LD with the others may be driving the observed association with the clinical outcome. Less LD is present in IL-6 which may indicate that several SNPs independent of each other may be driving the association of the gene with the clinical outcome of interest [Figure 2].

### 3.3. Single-SNP Analyses

**Figure 3:**
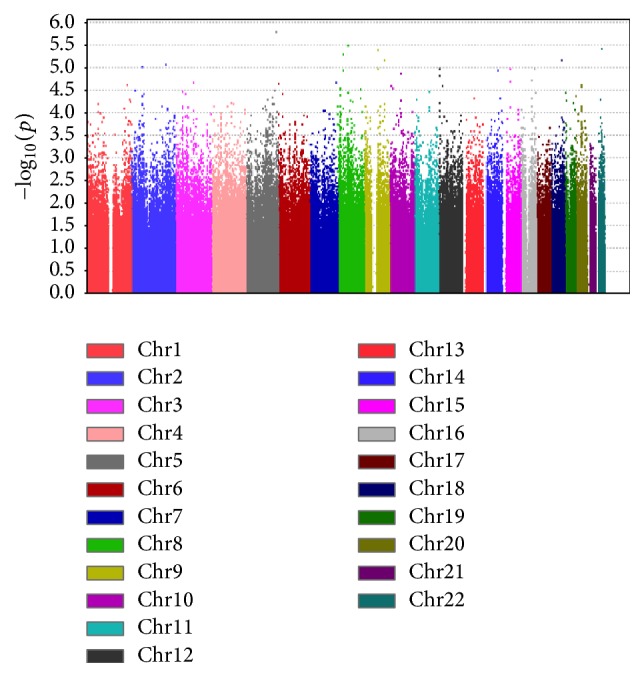
Manhattan plot summarizing the genome-wide association results. Smallest *p* = 1.62*e* − 6 (adjusted *p* = 0.328).

We also conducted separate single-SNP analyses similar to those above, putting only one SNP at a time in the full model. For the 150 candidate gene SNPs, we adjusted for population stratification and multiple testing of 150 SNPs (Supplementary Table  2). We conducted genome-wide analyses corrected for multiple testing, both with and without adjustment for population stratification (Figure 3). As expected given the small sample size, no SNP reached statistical significance in any of these analyses, given the less powerful SNP-based testing strategy, with its stricter multiple testing penalty.

The smallest raw* p* value of 1.62 × 10^−6^ (adjusted* p* = 0.328) corresponded to SNP rs2303135 on chromosome 5.

## 4. Discussion

We used a statistical methodology designed for small samples to confirm the finding of Duggal et al. demonstrating a significant association between spontaneous resolution of HCV infection and the* HLA-DQB1* region [9]. Their results identified an association between HCV clearance and single SNPs in the gene; our results demonstrate a genetic association that reflects a contribution from one or more SNPs in the gene region. In addition, we identified an association with another gene, encoding the cytokine interleukin 6 (IL-6), which is novel [22].

IL-6 is known as a marker of host inflammatory response and has been thought of as a mediator of innate immunity via its receptor interactions with gp130 and resulting proinflammatory transcriptional activity via both the JAK/STAT pathway and extracellular-signal-regulated kinase/mitogen-activated protein kinase (ERK/MAPK) pathways [23]. More recently, IL-6 has been shown to be active in cell-mediated immunity as an effector molecule that promotes differentiation of T-helper 17 (Th-17) cells which use interleukin-17 to increase transcription of proinflammatory genes [24]. Several studies have demonstrated that serum IL-6 levels are significantly elevated in HCV infected patients as compared to healthy controls [25–28]. Although one study showed significantly lower levels of* IL-6* gene expression, the vast majority of studies demonstrate significantly higher cytokine levels in HCV patients [29]. Relatively higher IL-6 elevations have been associated with patients with treatment failure versus successful treatment [26, 30].

The* IL-6 *−174 G/C SNP (rs1800795) in the* IL-6* promoter region, although not assayed in this study, has been associated with HCV treatment response and differential allele expression among races [31–33]. However, controversy exists regarding whether differential allele expression is associated with favorable treatment response. A possible confounder may be varying prevalence of other neighboring IL-6 promoter mutations (including rs1800796 and rs1800797), which may explain differences noted between studies to date [34–36]. Other studies suggest that the rs1800795 polymorphism is directly correlated with HCV spontaneous clearance [22]. Another study has demonstrated that this SNP may be important in autoimmune disease pathogenesis [37].

Our whole-gene analysis may have identified a previously undetected association of* IL-6* with spontaneous clearance by testing the total combined effect of multiple SNPs which are not in linkage disequilibrium with each other, each with a relatively small effect, and are therefore undetectable in a single-SNP-based test, especially when subjected to a genome-wide multiple testing correction. Thus, it can be possible by examining the gene as a whole to identify novel genic associations with a clinical outcome that might elude single-SNP analysis.

Our study is limited by the small sample, but this limitation can be partly overcome by restricting analysis to a preselected set of candidate genes with* a priori* evidence and by testing genes rather than SNPs in order to reduce the number of tests and increase power. Large samples are expensive and sometimes unavailable, if few patients meet appropriate inclusion/exclusion criteria. In fact, it is sometimes impossible to obtain a larger sample except by introducing greater heterogeneity into the study population, which might sometimes reduce power to the point of offsetting any advantage of sample size. The present strategy may also be more successful when a gene has many individual SNPs, each making a small contribution to the phenotype.

Race was significantly different between the chronic infection and spontaneous clearer groups and was missing for 16 patients. The most common reasons for missing data were patients listing race as Hispanic (which was coded as an ethnicity for the purposes of this study) and not defining any other racial category. This may have contributed to the difference as there was more missing data in the spontaneous clearance group. The other reason may be a true reflection of racial differences in spontaneous clearance as prior studies have also shown a higher rate of persistent HCV infection in African ancestry populations [9].

Some SNPs, possibly associated with spontaneous clearance in prior literature, were not included on the chip array. Rs4272729 (in* HLA-DQB1* gene region), rs1800795, rs1800796, and rs1800797 (in* IL-6* gene region) were not included in either the Illumina 610 or 660W Quad chip used to assay sample DNA. Although included in the analysis,* IL-28B* SNPs rs12979860 and rs8099917 were not found to be significantly associated with spontaneous viral clearance. In addition, the* IL-28B* gene as a whole was not found to be significantly associated with spontaneous clearance in this analysis. This may be due to an insufficient number of patients to demonstrate a difference, as well as the small number of SNPs assayed, as the association of SNPs in the* IL-28B* region with viral clearance is strongly suggested in prior literature [4–7, 9].

Understanding the immunologic host factors that predict clearance of HCV is important in estimating treatment prognosis for different patients. However, it may also be of critical importance in the future for selecting antivirals for treatment, for vaccine development, and for the development of more potent antivirals. Additionally, understanding the genes involved in the clearance of HCV may provide a clearer understanding of the human immune response to viral pathogens and disease. Our whole-gene analytic strategy identified a previously unreported association of IL-6 with spontaneous clearance of HCV infection. We also confirmed an earlier finding that HLA-DQB1 is associated with spontaneous clearance.

## Supplementary Material

Supplementary materials include the following: quantile-quantile plots of the test statistics of 309,470 null SNPs before and after correction for population genetic structure using the first ancestry eigenvector; LD maps of all analyzed gene regions; genotype counts and frequencies of SNPs within HLA-DQB1, IL-6, and IL28B; and Step-down permutation testing applied to univariate analyses of 150 SNPs.

## Figures and Tables

**Table 1:** Candidate genes selection.

Candidate genes associated with HCV spontaneous clearance or response to therapy			
Gene symbol	Chromosome location	Gene name	Number of SNPs in analysis
IL28B	19q13.13	Interleukin 28B (encoding IFN-gamma-3)	4
SOCS3	17q25.3	Suppressor of cytokine signaling 3	16
SOCS1	16p13.13	Suppressor of cytokine signaling 1	2
IL-10	1q31-q32	Interleukin 10	15
IL-18	11q22.2-q22.3	Interleukin 18	6
TNF-alpha	6p21.3	Tumor necrosis factor alpha	1
TGF-beta-1	19q13.1	Transforming growth factor, beta 1	4
IL-6	7p21	Interleukin 6	42
CTLA-4	2q33	Cytotoxic T-lymphocyte-associated protein 4	7
IFNG	12q14	Interferon, gamma	8
HLA genes:			
HLA-DRB1	6p21.3	Major histocompatibility complex, class II, DR beta 1	5
HLA-DQB1	6p21.3	Major histocompatibility complex, class II, DQ beta 1	40

**Table 2:** Demographic and clinical characteristics of subjects.

	Total *N* = 157	Spontaneous resolution *N* = 62 (39%)	Chronic infection *N* = 95 (61%)	*p* value (Chi-squared test unless otherwise noted)
*Age*, *mean (SD) [range], years*	55.9 (8.2) [25, 81]	55.7 (8.7) [25, 77]	55.9 (7.8) [35, 81]	0.87 (two-sample *t*-test)
*Gender, number (%)*				0.44
Male	132 (84%)	54 (87%)	78 (82%)	
Female	23 (15%)	8 (13%)	15 (16%)	
Trans	2 (1%)	0	2 (2%)	
*Ethnicity, number (%)*				0.53
Not Hispanic	126 (80%)	48 (77%)	78 (82%)	
Hispanic	20 (13%)	10 (16%)	10 (11%)	
Mixed	6 (4%)	3 (5%)	3 (3%)	
Missing	5 (3%)	1 (2%)	4 (4%)	
*Self-identified race, number (%)*				0.01
Caucasian	84 (54%)	36 (58%)	48 (51%)	
Black	43 (27%)	8 (13%)	35 (37%)	
Other or mixed	14 (9%)	7 (11%)	7 (7%)	
Missing	16 (10%)	11 (18%)	5 (5%)	
*HIV status, number (%)*				0.26
Infected	42 (27%)	13 (21%)	29 (31%)	
Not infected	115 (73%)	49 (79%)	66 (69%)	
*HCG genotype, number (%)*				NA
1	NA	NA	67 (71%)	
2	NA	NA	9 (10%)	
3	NA	NA	11 (12%)	
4	NA	NA	4 (4%)	
Unknown	NA	NA	4 (4%)	
*HCV treatment, number (%)*				NA
SVR	NA	NA	23 (24%)	
NR	NA	NA	7 (7%)	
Not treated	NA	NA	65 (68%)	

NA: not available.

**Table 3:** Likelihood ratio test (LRT) results comparing full models (consisting of all SNPs for the gene) and null models after controlling for HIV infection.

Not controlling for HIV			Controlling for HIV		
Gene name	LRT *p* value	5% significance threshold^*∗*^	Gene name	LRT *p* value	5% significance threshold^*∗*^
HLA-DQB1	1.76*∗*10^−5^	0.0006	HLA-DQB1	1.84*∗*10^−5^	0.0005
IL-6	0.0007	0.0117	IL-6	0.0008	0.0110
IL-28B	0.1700	0.0347	IL28B	0.1600	0.0324
IL-10	0.1987	0.0697	IL-10	0.2603	0.0645
CTLA-4	0.4514	0.1124	CTLA4	0.4305	0.1059
HLA-DRB1	0.4981	0.1661	HLA-DRB1	0.4759	0.1602
SOCS1	0.5313	0.2264	SOCS1	0.5071	0.2195
TNF	0.6957	0.2952	TNF	0.6508	0.2888
IFNG	0.7391	0.3755	SOCS3	0.7475	0.3675
TGF-beta-1	0.7590	0.4677	TGF-beta-1	0.8025	0.4548
SOCS3	0.7742	0.5738	IFNG	0.8027	0.5630
IL-18	0.9567	0.7027	IL-18	0.9649	0.7037

^*∗*^5% significance thresholds derived from permutation distributions (unstratified). A gene reaches statistical significance under the step-down procedure when the observed LRT *p* values for it and all more significant genes are less than their corresponding 5% thresholds.
