# Supplementary material for: Genetic Variation in the IL-6 and HLA-DQB1 Genes Is Associated with Spontaneous Clearance of Hepatitis C Virus Infection
Source: J Immunol Res. 2016 Jun 2;2016:6530436. doi: 10.1155/2016/6530436 (PMC4909898; doi:10.1155/2016/6530436)
Supplement: Supplementary file 1 — Supplementary materials include the following: quantile-quantile plots of the test statistics of 309,470 null SNPs before and after correction for population genetic structure using the first ancestry eigenvector; LD maps of all analyzed gene regions; genotype counts and frequencies of SNPs within HLA-DQB1, IL-6, and IL28B; and Step-down permutation testing applied to univariate analyses of 150 SNPs. [file 6530436.f1.pdf]

Supplemental

Supplementary Figure 1. Quantile-quantile plots of the test statistics of 309,470 null SNPs before (left) and after (right) correction for population genetic structure using the first ancestry eigenvector.

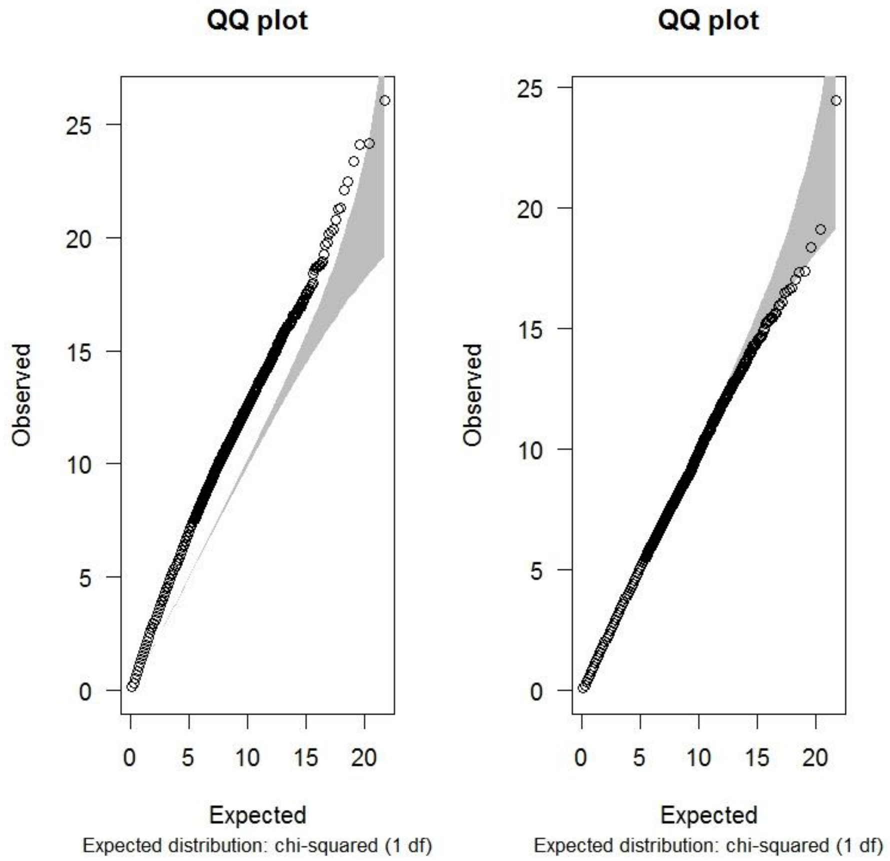

Supplementary Figure 2. LD maps

(A) IL28B

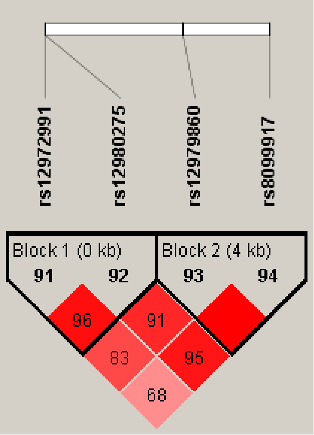

(B) SOCS1, TNF, HLA-DRB1, TGFB1

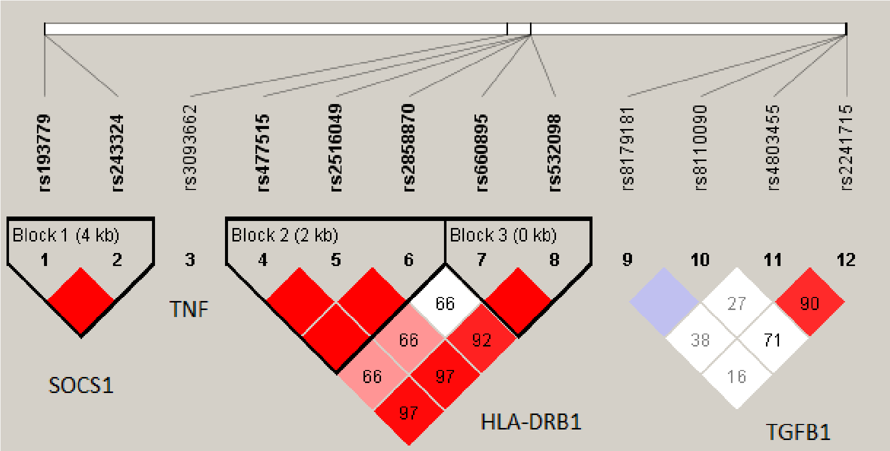

(C) IFNG, SOCS3

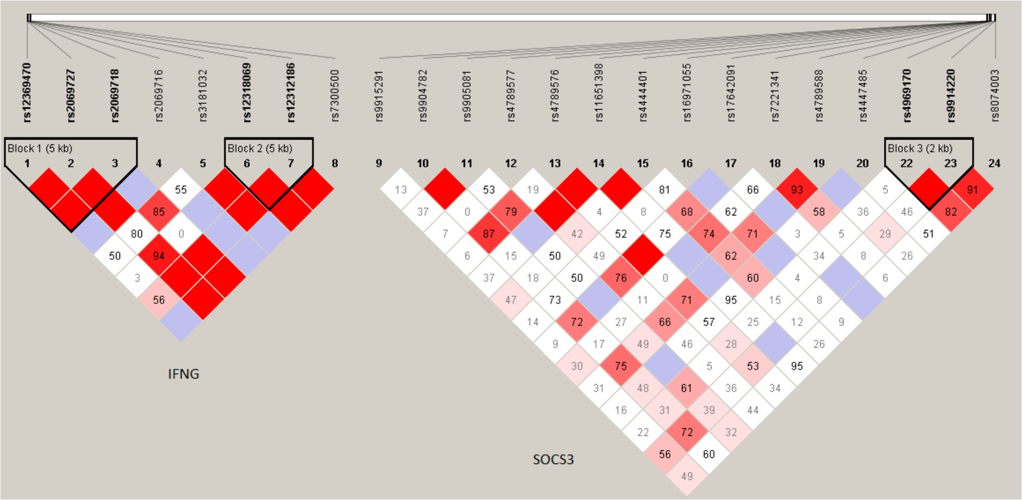

(D) IL18, CTLA4

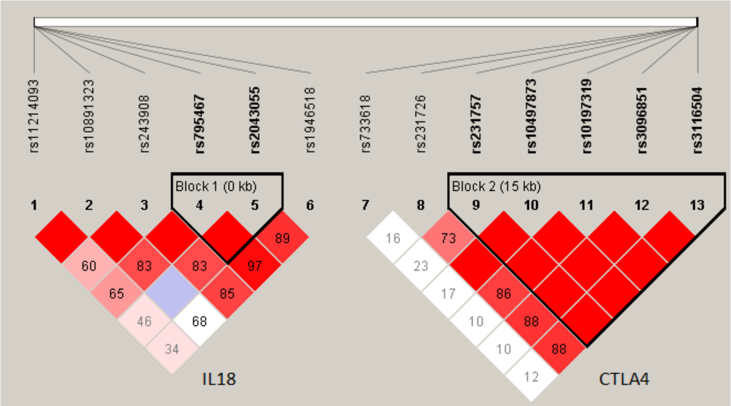

(E) IL10

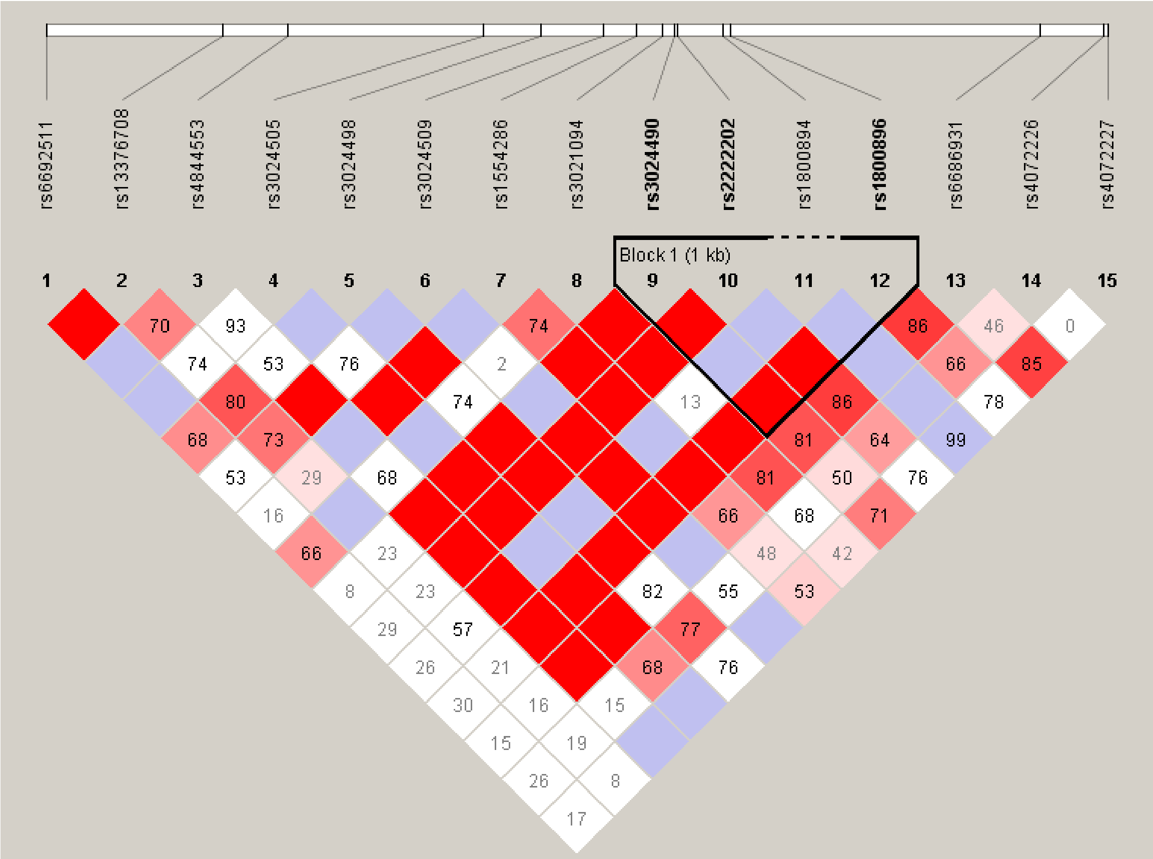
rs6692511
rs13376708
rs4844553
rs3024505
rs3024498
rs3024509
rs1554286
rs3021094
rs3024490
rs2222202
rs1800894
rs1800896
rs6686931
rs4072226
rs4072227
Block 1 (1 kb)
1
2
3
4
5
6
7
8
9
10
11
12
13
14
15

Supplementary Table 1A. Genotype counts and frequencies of SNPs within HLA-DQB1

SNP	Alleles	Spontaneous Resolution n = 62	Chronic HCV Infection n = 95	Logistic Regression Unadjusted p-value
rs1063355	AA<br>Aa<br>aa<br>missing	22 (35%)<br>31 (50%)<br>9 (15%)<br>0	50 (53%)<br>37 (39%)<br>8 (8%)<br>0	0.6112
rs9275141	AA<br>Aa<br>aa<br>missing	23 (37%)<br>28 (45%)<br>10 (16%)<br>1 (2%)	27 (28%)<br>43 (45%)<br>25 (26%)<br>0	0.2093
rs7755224	AA<br>Aa<br>aa<br>missing	48 (77%)<br>14 (23%)<br>0 (0%)<br>0	75 (79%)<br>18 (19%)<br>2 (2%)<br>0	0.9927
rs2856683	AA<br>Aa<br>aa<br>missing	33 (53%)<br>22 (35%)<br>4 (6%)<br>3 (5%)	53 (56%)<br>34 (36%)<br>6 (6%)<br>2 (2%)	0.7487
rs7774434	AA<br>Aa<br>aa<br>missing	24 (39%)<br>27 (44%)<br>10 (16%)<br>1 (2%)	33 (35%)<br>50 (53%)<br>12 (13%)<br>0	0.6408
rs7775228	AA<br>Aa<br>aa<br>missing	47 (76%)<br>12 (19%)<br>2 (3%)<br>1 (2%)	68 (72%)<br>25 (26%)<br>1 (1%)<br>1 (1%)	0.6964
rs9275224	AA<br>Aa<br>aa<br>missing	27 (44%)<br>27 (44%)<br>8 (13%)<br>0	24 (25%)<br>51 (54%)<br>20 (21%)<br>0	0.0353
rs5000634	AA<br>Aa<br>aa<br>missing	15 (24%)<br>30 (48%)<br>17 (27%)<br>0	34 (36%)<br>47 (49%)<br>14 (15%)<br>0	0.0783
rs6457617	AA<br>Aa<br>aa<br>missing	25 (40%)<br>28 (45%)<br>9 (15%)<br>0	22 (23%)<br>53 (56%)<br>20 (21%)<br>0	0.0720
rs2647012	AA<br>Aa<br>aa	32 (52%)<br>25 (40%)<br>5 (8%)	39 (41%)<br>41 (43%)<br>15 (16%)	0.1900

	missing	0	0	
rs9357152	AA<br>Aa<br>aa<br>missing	28 (45%)<br>28 (45%)<br>6 (10%)<br>0	50 (53%)<br>42 (44%)<br>3 (3%)<br>0	0.1753
rs10484561	AA<br>Aa<br>aa<br>missing	48 (77%)<br>14 (23%)<br>0<br>0	75 (79%)<br>18 (19%)<br>2 (2%)<br>0	0.9927
rs9275312	AA<br>Aa<br>aa<br>missing	39 (63%)<br>19 (31%)<br>4 (6%)<br>0	69 (73%)<br>23 (24%)<br>3 (3%)<br>0	0.2563
rs9275313	AA<br>Aa<br>aa<br>missing	47 (76%)<br>15 (24%)<br>0<br>0	77 (81%)<br>13 (14%)<br>4 (4%)<br>1 (1%)	0.7300
rs1794282	AA<br>Aa<br>aa<br>missing	56 (90%)<br>6 (10%)<br>0<br>0	80 (84%)<br>15 (16%)<br>0<br>0	0.0745
rs2856725	AA<br>Aa<br>aa<br>missing	30 (48%)<br>25 (40%)<br>5 (8%)<br>2 (3%)	39 (41%)<br>41 (43%)<br>15 (16%)<br>0	0.2735
rs9275328	AA<br>Aa<br>aa<br>missing	37 (60%)<br>18 (29%)<br>4 (6%)<br>3 (5%)	67 (71%)<br>23 (24%)<br>3 (3%)<br>2 (2%)	0.2648
rs2647044	AA<br>Aa<br>aa<br>missing	50 (81%)<br>5 (8%)<br>1 (2%)<br>6 (10%)	66 (69%)<br>19 (20%)<br>7 (7%)<br>3 (3%)	0.0052
rs9275371	AA<br>Aa<br>aa<br>missing	28 (45%)<br>22 (35%)<br>7 (11%)<br>5 (8%)	50 (53%)<br>36 (38%)<br>6 (6%)<br>3 (3%)	0.4246
rs9275374	AA<br>Aa<br>aa<br>missing	28 (45%)<br>27 (44%)<br>7 (11%)<br>0	50 (53%)<br>38 (40%)<br>6 (6%)<br>1 (1%)	0.2951
rs9275383	AA<br>Aa<br>aa	43 (69%)<br>16 (26%)<br>0	76 (80%)<br>16 (17%)<br>0	0.2721

	missing	3 (5%)	3 (3%)	
rs9275388	AA<br>Aa<br>aa<br>missing	28 (45%)<br>27 (44%)<br>7 (11%)<br>0	49 (52%)<br>39 (41%)<br>6 (6%)<br>1 (1%)	0.3560
rs9275390	AA<br>Aa<br>aa<br>missing	28 (45%)<br>27 (44%)<br>7 (11%)<br>0	50 (53%)<br>39 (41%)<br>6 (6%)<br>0	0.3206
rs9275393	AA<br>Aa<br>aa<br>missing	28 (45%)<br>27 (44%)<br>7 (11%)<br>0	50 (53%)<br>39 (41%)<br>6 (6%)<br>0	0.3206
rs2647050	AA<br>Aa<br>aa<br>missing	22 (35%)<br>32 (52%)<br>8 (13%)<br>0	38 (40%)<br>44 (46%)<br>13 (14%)<br>0	0.8345
rs9275406	AA<br>Aa<br>aa<br>missing	28 (45%)<br>27 (44%)<br>7 (11%)<br>0	50 (53%)<br>39 (41%)<br>6 (6%)<br>0	0.3206
rs2858308	AA<br>Aa<br>aa<br>missing	54 (87%)<br>8 (13%)<br>0<br>0	74 (78%)<br>20 (21%)<br>1 (1%)<br>0	0.0995
rs9275407	AA<br>Aa<br>aa<br>missing	28 (45%)<br>27 (44%)<br>7 (11%)<br>0	50 (53%)<br>38 (40%)<br>6 (6%)<br>1 (1%)	0.2951
rs9275418	AA<br>Aa<br>aa<br>missing	29 (47%)<br>26 (42%)<br>7 (11%)<br>0	50 (53%)<br>39 (41%)<br>6 (6%)<br>0	0.4338
rs2856718	AA<br>Aa<br>aa<br>missing	22 (35%)<br>32 (52%)<br>8 (13%)<br>0	38 (40%)<br>44 (46%)<br>13 (14%)<br>0	0.8345
rs2856717	AA<br>Aa<br>aa<br>missing	32 (52%)<br>25 (40%)<br>5 (8%)<br>0	40 (42%)<br>41 (43%)<br>14 (15%)<br>0	0.2533
rs2858305	AA<br>Aa<br>aa	32 (52%)<br>25 (40%)<br>5 (8%)	40 (42%)<br>41 (43%)<br>14 (15%)	0.2533

	missing	0	0	
rs9275424	AA<br>Aa<br>aa<br>missing	26 (42%)<br>25 (40%)<br>7 (11%)<br>4 (6%)	49 (52%)<br>39 (41%)<br>6 (6%)<br>1 (1%)	0.2810
rs9275425	AA<br>Aa<br>aa<br>missing	28 (45%)<br>26 (42%)<br>7 (11%)<br>1 (2%)	50 (53%)<br>39 (41%)<br>6 (6%)<br>0	0.3498
rs9275427	AA<br>Aa<br>aa<br>missing	28 (45%)<br>27 (44%)<br>7 (11%)<br>0	50 (53%)<br>39 (41%)<br>6 (6%)<br>0	0.3206
rs2856705	AA<br>Aa<br>aa<br>missing	54 (87%)<br>8 (13%)<br>0<br>0	74 (78%)<br>19 (20%)<br>1 (1%)<br>1 (1%)	0.1323
rs9275428	AA<br>Aa<br>aa<br>missing	28 (45%)<br>27 (44%)<br>7 (11%)<br>0	50 (53%)<br>39 (41%)<br>6 (6%)<br>0	0.3206
rs13192471	AA<br>Aa<br>aa<br>missing	42 (68%)<br>17 (27%)<br>3 (5%)<br>0	67 (71%)<br>26 (27%)<br>2 (2%)<br>0	0.4603
rs1794275	AA<br>Aa<br>aa<br>missing	40 (65%)<br>15 (24%)<br>5 (8%)<br>2 (3%)	62 (65%)<br>28 (29%)<br>3 (3%)<br>2 (2%)	0.5559
rs9275439	AA<br>Aa<br>aa<br>missing	28 (45%)<br>27 (44%)<br>7 (11%)<br>0	50 (53%)<br>39 (41%)<br>5 (5%)<br>1 (1%)	0.2770

Supplementary Table 1B. Genotype counts and frequencies of SNPs within IL6

SNP	Alleles	Spontaneous Resolution n = 62	Chronic HCV Infection n = 95	Logistic Regression Unadjusted p-value
rs2961285	AA<br>Aa<br>aa<br>missing	18 (29%)<br>33 (53%)<br>11 (18%)<br>0	28 (29%)<br>43 (45%)<br>24 (25%)<br>0	0.6558
rs7793163	AA<br>Aa<br>aa<br>missing	49 (79%)<br>13 (21%)<br>0<br>0	74 (78%)<br>18 (19%)<br>(3 (3%)<br>(0	0.9107
rs12535797	AA<br>Aa<br>aa<br>missing	55 (89%)<br>7 (11%)<br>0<br>0	78 (82%)<br>14 (15%)<br>1 (1%)<br>2	0.1637
rs4722164	AA<br>Aa<br>aa<br>missing	48 (77%)<br>13 (21%)<br>1 (2%)<br>0	74 (78%)<br>18 (19%)<br>3 (3%)<br>0	0.6217
rs2961294	AA<br>Aa<br>aa<br>missing	16 (26%)<br>31 (50%)<br>15 (24%)<br>0	29 (31%)<br>41 (43%)<br>25 (26%)<br>0	0.1737
rs1989838	AA<br>Aa<br>aa<br>missing	27 (44%)<br>28 (45%)<br>7 (11%)<br>0	49 (52%)<br>40 (42%)<br>6 (6%)<br>0	0.1696
rs2905321	AA<br>Aa<br>aa<br>missing	17 (27%)<br>31 (50%)<br>14 (23%)<br>0	31 (33%)<br>37 (39%)<br>27 (28%)<br>0	0.4200
rs2905325	AA<br>Aa<br>aa<br>missing	53 (85%)<br>9 (15%)<br>0<br>0	77 (81%)<br>15 (16%)<br>3 (3%)<br>0	0.6346
rs4719709	AA<br>Aa<br>aa<br>missing	33 (53%)<br>26 (42%)<br>3 (5%)<br>0	46 (48%)<br>42 (44%)<br>7 (7%)<br>0	0.1936

rs13225099	AA<br>Aa<br>aa<br>missing	37 (60%)<br>22 (35%)<br>3 (5%)<br>0	50 (53%)<br>39 (41%)<br>6 (6%)<br>0	0.2727
rs1476482	AA<br>Aa<br>aa<br>missing	47 (76%)<br>12 (19%)<br>3 (5%)<br>0	63 (66%)<br>25 (26%)<br>7 (7%)<br>0	0.6964
rs1476483	AA<br>Aa<br>aa<br>missing	48 (77%) (<br>13 (21%)<br>1 (2%)<br>0	63 (66%)<br>31 (33%)<br>1 (1%)<br>0	0.0927
rs2106549	AA<br>Aa<br>aa<br>missing	54 (87%)<br>8 (13%)<br>0<br>0	78 (82%)<br>13 (14%)<br>3 (3%)<br>1	0.5406
rs7802442	AA<br>Aa<br>aa<br>missing	25 (40%)<br>31 (50%)<br>6 (10%)<br>0	30 (32%)<br>41 (43%)<br>24 (25%)<br>0	0.2925
rs4722166	AA<br>Aa<br>aa<br>missing	40 (65%)<br>20 (32%)<br>2 (3%)<br>0	51 (54%)<br>37 (39%)<br>7 (7%)<br>0	0.0137
rs6949149	AA<br>Aa<br>aa<br>missing	48 (77%)<br>13 (21%)<br>1 (2%)<br>0	77 (81%)<br>15 (16%)<br>3 (3%)<br>0	0.4693
rs6954897	AA<br>Aa<br>aa<br>missing	26 (42%)<br>32 (52%)<br>4 (6%)<br>0	36 (38%)<br>41 (43%)<br>18 (19%)<br>0	0.0063
rs6946864	AA<br>Aa<br>aa<br>missing	23 (37%)<br>29 (47%)<br>10 (16%)<br>0	47 (49%)<br>28 (29%)<br>20 (21%)<br>0	0.0144
rs10156056	AA<br>Aa<br>aa<br>missing	37 (60%)<br>23 (37%)<br>1 (2%)<br>1	68 (72%)<br>25 (26%)<br>2 (2%)<br>0	0.0849

rs7776857	AA<br>Aa<br>aa<br>missing	42 (68%)<br>18 (29%)<br>2 (3%)<br>0	55 (58%)<br>34 (36%)<br>6 (6%)<br>0	0.0146
rs7801617	AA<br>Aa<br>aa<br>missing	35 (56%)<br>25 (40%)<br>2 (3%)<br>0	66 (69%)<br>22 (23%)<br>7 (7%)<br>0	0.0592
rs7805828	AA<br>Aa<br>aa<br>missing	22 (35%)<br>28 (45%)<br>12 (19%)<br>0	34 (36%)<br>45 (47%)<br>16 (17%)<br>0	0.9531
rs1880242	AA<br>Aa<br>aa<br>missing	26 (42%)<br>30 (48%)<br>6 (10%)<br>0	36 (38%)<br>44 (46%)<br>15 (16%)<br>0	0.0973
rs10499563	AA<br>Aa<br>aa<br>missing	30 (48%)<br>30 (48%)<br>2 (3%)<br>0	55 (58%)<br>33 (35%)<br>7 (7%)<br>0	0.5727
rs2056576	AA<br>Aa<br>aa<br>missing	24 (39%)<br>32 (52%)<br>6 (10%)<br>0	42 (44%)<br>46 (48%)<br>7 (7%)<br>0	0.4245
rs2069837	AA<br>Aa<br>aa<br>missing	47 (76%)<br>15 (24%)<br>0<br>0	78 (82%)<br>15 (16%)<br>2 (2%)<br>0	0.4317
rs2066992	AA<br>Aa<br>aa<br>missing	48 (77%)<br>14 (23%)<br>0<br>0	77 (81%)<br>17 (18%)<br>1 (1%)<br>0	0.5638
rs1554606	AA<br>Aa<br>aa<br>missing	31 (50%)<br>27 (44%)<br>4 (6%)<br>0	36 (38%)<br>46 (48%)<br>13 (14%)<br>0	0.0426
rs10242595	AA<br>Aa<br>aa<br>missing	15 (24%)<br>32 (52%)<br>15 (24%)<br>0	44 (46%)<br>33 (35%)<br>17 (18%)<br>1	0.0017

rs11766273	AA<br>Aa<br>aa<br>missing	58 (94%)<br>4 (6%)<br>0<br>0	85 (89%)<br>10 (11%)<br>0<br>0	0.2311
rs7802277	AA<br>Aa<br>aa<br>missing	29 (47%)<br>27 (44%)<br>6 (10%)<br>0	65 (68%)<br>23 (24%)<br>7 (7%)<br>0	0.0093
rs7801406	AA<br>Aa<br>aa<br>missing	39 (63%)<br>21 (34%)<br>2 (3%)<br>0	50 (53%)<br>36 (38%)<br>9 (9%)<br>0	0.0030
rs6461666	AA<br>Aa<br>aa<br>missing	26 (42%)<br>25 (40%)<br>11 (18%)<br>0	51 (54%)<br>34 (36%)<br>10 (11%)<br>0	0.0166
rs6461667	AA<br>Aa<br>aa<br>missing	39 (63%)<br>21 (34%)<br>2 (3%)<br>0	50 (53%)<br>36 (38%)<br>9 (9%)<br>0	0.0030
rs4722178	AA<br>Aa<br>aa<br>missing	24 (39%)<br>28 (45%)<br>9 (15%)<br>1	15 (16%)<br>53 (56%)<br>26 (27%)<br>1	0.0047
rs10229203	AA<br>Aa<br>aa<br>missing	41 (66%)<br>20 (32%)<br>1 (2%)<br>0	80 (84%)<br>12 (13%)<br>3 (3%)<br>0	0.0199
rs10229457	AA<br>Aa<br>aa<br>missing	16 (26%)<br>29 (47%)<br>17 (27%)<br>0	38 (40%)<br>32 (34%)<br>25 (26%)<br>0	0.0109
rs7796691	AA<br>Aa<br>aa<br>missing	31 (50%)<br>26 (42%)<br>5 (8%)<br>0	73 (77%)<br>19 (20%)<br>3 (3%)<br>0	0.0013
rs7782803	AA<br>Aa<br>aa<br>missing	47 (76%)<br>14 (23%)<br>1 (2%)<br>0	64 (67%)<br>24 (25%)<br>7 (7%)<br>0	0.6511

rs7793526	AA<br>Aa<br>aa<br>missing	20 (32%)<br>29 (47%)<br>13 (21%)<br>0	47 (49%)<br>38 (40%)<br>10 (11%)<br>0	0.0724
rs1608555	AA<br>Aa<br>aa<br>missing	20 (32%)<br>26 (42%)<br>15 (24%)<br>1	46 (48%)<br>37 (39%)<br>10 (11%)<br>2	0.0473
rs10950917	AA<br>Aa<br>aa<br>missing	38 (61%)<br>21 (34%)<br>3 (5%)<br>0	48 (51%)<br>38 (40%)<br>9 (9%)<br>0	0.0653

Supplementary Table 1C. Genotype counts and frequencies of SNPs within IL28B

SNP	Alleles	Spontaneous Resolution n = 62	Chronic HCV Infection n = 95	Logistic Regression Unadjusted p-value
rs12972991	AA	46 (74%)	59 (62%)	0.0206
	Aa	15 (24%)	30 (32%)	
	aa	1 (2%)	6 (6%)	
	missing	0	0	
rs12979860	AA	34 (55%)	32 (34%)	0.0896
	Aa	20 (32%)	47 (49%)	
	aa	6 (10%)	16 (17%)	
	missing	2 (3%)	0	
rs12980275	AA	35 (56%)	31 (33%)	0.0324
	Aa	22 (35%)	47 (49%)	
	aa	5 (8%)	17 (18%)	
	missing	0	0	
rs8099917	AA	49 (79%)	71 (75%)	0.2826
	Aa	11 (18%)	21 (22%)	
	aa	2 (3%)	3 (3%)	
	missing	0	0	

Supplementary Table 2. Step-down permutation testing applied to univariate analyses of 150 SNPs: *p*-values and 5% significance thresholds derived from permutation distributions.

Gene Name	SNP Name	*p*-value	5% Significance Threshold
IL6	rs7796691	0.001295594	0.000424816
	rs10242595	0.001658856	0.001998481
	rs7801406	0.002951262	0.003353515
	rs6461667	0.002951262	0.005798252
	rs4722178	0.00471515	0.008469925
HLA-DQB1	rs2647044	0.005179592	0.010640514
IL6	rs6954897	0.0062929	0.013691308
	rs7802277	0.009344	0.016350194
	rs10229457	0.010906335	0.019724857
	rs4722166	0.013709627	0.022196036
	rs6946864	0.014424173	0.025133073
	rs7776857	0.014585638	0.028587283
	rs6461666	0.016597254	0.03290528
	rs10229203	0.019889686	0.036009737
IL28B	rs12972991	0.020637531	0.037851262
IL10	rs4072226	0.025948728	0.04179366
IL28B	rs12980275	0.032370623	0.046516024
HLA-DQB1	rs9275224	0.035265809	0.049985218
IL6	rs1554606	0.042552004	0.053198974
	rs1608555	0.04733872	0.055736244
	rs7801617	0.059222031	0.060487771
	rs10950917	0.065254838	0.064963748
IFNG	rs7300500	0.069176604	0.067961175
HLA-DQB1	rs6457617	0.07198693	0.071236136
IL6	rs7793526	0.07239997	0.075657335
HLA-DQB1	rs1794282	0.07452235	0.081084643
SOCS3	rs9904782	0.07572546	0.082480017
HLA-DQB1	rs5000634	0.078266814	0.088253296
IL6	rs10156056	0.084928299	0.094930399
IL28B	rs1297986	0.089619078	0.096806374
IL6	rs1476483	0.092702583	0.101076642
	rs1880242	0.097253147	0.104670071
HLA-DQB1	rs2858308	0.099463921	0.109041357
	rs2856705	0.132304898	0.115149252
IL10	rs1800896	0.141556585	0.120188111

HLA-DRB1	rs2858870	0.152846222	0.124542626
IL6	rs12535797	0.163707402	0.128573644
IL10	rs3024490	0.168290547	0.133058213
IL6	rs1989838	0.169551692	0.140056139
	rs2961294	0.173689338	0.143520418
HLA-DQB1	rs9357152	0.175292647	0.148806491
	rs2647012	0.189971106	0.153859061
IL6	rs4719709	0.193559811	0.158835489
IL10	rs3024498	0.194293291	0.16526242
HLA-DQB1	rs9275141	0.209292633	0.170365376
IL10	rs2222202	0.215392829	0.175996641
IL6	rs11766273	0.231060255	0.182991836
IL10	rs4072227	0.235401961	0.18929008
HLA-DQB1	rs2856717	0.253339991	0.195643162
	rs2858305	0.253339991	0.201414165
	rs9275312	0.25628026	0.206559346
	rs9275328	0.264821022	0.215186952
	rs9275383	0.272062539	0.220995039
IL6	rs13225099	0.27272189	0.22599771
HLA-DQB1	rs2856725	0.273487607	0.233012139
	rs9275439	0.277021557	0.238032872
HLA-DRB1	rs532098	0.280665473	0.243465492
HLA-DQB1	rs9275424	0.281049773	0.250054
IL28B	rs8099917	0.282593607	0.255983217
IL10	rs6692511	0.284413374	0.258247737
IFNG	rs2069727	0.290727497	0.263896557
IL6	rs7802442	0.292531693	0.270086689
HLA-DQB1	rs9275374	0.295051663	0.276904216
	rs9275407	0.295051663	0.28363915
IFNG	rs2069716	0.304227598	0.289563808
HLA-DQB1	rs9275390	0.320561531	0.29256828
	rs9275393	0.320561531	0.298895928
	rs9275406	0.320561531	0.310345206
	rs9275427	0.320561531	0.318043853
	rs9275428	0.320561531	0.326169435
SOCS3	rs4969170	0.326312938	0.329998686
IL10	rs1554286	0.338019606	0.337286673
CTLA4	rs10197319	0.339650595	0.343004728
HLA-DQB1	rs9275425	0.349774133	0.351130007
CTLA4	rs231757	0.353991648	0.355875747

HLA-DQB1	rs9275388	0.355970967	0.362527245
CTLA4	rs3096851	0.384889529	0.372029815
TGFB1	rs4803455	0.390065937	0.379657177
IFNG	rs12318069	0.406925443	0.385307332
IL18	rs11214093	0.407891769	0.393303321
TGFB1	rs8110090	0.419548538	0.397513853
IL6	rs2905321	0.420016363	0.40541972
HLA-DRB1	rs477515	0.420887157	0.414301491
IL6	rs2056576	0.424503234	0.419976456
HLA-DQB1	rs9275371	0.424606885	0.426766822
TGFB1	rs2241715	0.428948349	0.433463862
IL6	rs2069837	0.431703257	0.437437397
HLA-DQB1	rs9275418	0.433766926	0.447072476
SOCS3	rs7221341	0.437658958	0.453255839
HLA-DQB1	rs13192471	0.460255346	0.463080741
HLA-DRB1	rs2516049	0.464577715	0.470253348
IL6	rs6949149	0.469292213	0.474381917
SOCS1	rs243324	0.493153314	0.484426413
IFNG	rs2069718	0.496412743	0.490868931
CTLA4	rs3116504	0.507763054	0.497425146
SOCS3	rs4447485	0.508406434	0.505689156
	rs4969168	0.512972802	0.513962975
	rs9914220	0.532129163	0.522385758
IL18	rs2043055	0.539877906	0.530759541
IL6	rs2106549	0.540614244	0.538492717
SOCS3	rs4789576	0.545021779	0.545641147
HLA-DQB1	rs1794275	0.555916178	0.554401401
IL6	rs2066992	0.563800812	0.563711963
IL10	rs4844553	0.570641513	0.569465272
IL6	rs10499563	0.57268103	0.575826026
SOCS3	rs16971055	0.580789343	0.581624466
	rs9915291	0.582768762	0.590312662
HLA-DQB1	rs1063355	0.611184732	0.597061569
IL6	rs4722164	0.621693163	0.605862865
SOCS3	rs9905081	0.630302034	0.613657187
IL6	rs2905325	0.634573224	0.62049716
HLA-DQB1	rs7774434	0.640764277	0.630613706
IL6	rs7782803	0.651091034	0.638288526
IL10	rs13376708	0.654430951	0.648340073
IL6	rs2961285	0.655752635	0.657353009

SOCS3	rs17642091	0.665249848	0.663802448
	rs4444401	0.683760745	0.670864932
IL6	rs1476482	0.696422869	0.67766323
HLA-DQB1	rs7775228	0.696424945	0.685993901
TNF	rs3093662	0.704348534	0.692355233
HLA-DQB1	rs9275313	0.729966945	0.702326267
	rs2856683	0.748742325	0.711597997
SOCS3	rs4789588	0.750951037	0.723150481
IFNG	rs12312186	0.761198009	0.730423781
SOCS3	rs4789577	0.773013808	0.736548142
HLA-DRB1	rs660895	0.780978726	0.746366369
IL18	rs243908	0.795416049	0.755026112
IL10	rs3024505	0.800648674	0.763840575
IL18	rs10891323	0.817008557	0.769688318
IFNG	rs3181032	0.818646628	0.778861619
HLA-DQB1	rs2647050	0.83450225	0.786700311
	rs2856718	0.83450225	0.795327495
IL18	rs1946518	0.850037602	0.805072943
SOCS3	rs8074003	0.851126633	0.816250043
SOCS1	rs193779	0.85301645	0.825070093
IL10	rs1800894	0.855939093	0.834437819
CTLA4	rs733618	0.875491412	0.845638403
IL10	rs3021094	0.900520058	0.855876601
IL6	rs7793163	0.910673973	0.862996257
SOCS3	rs11651398	0.917219397	0.873108087
IL10	rs6686931	0.919245025	0.881948512
IL18	rs795467	0.923049199	0.890637866
IL10	rs3024509	0.928900221	0.899320486
CTLA4	rs10497873	0.94141377	0.911675151
IL6	rs7805828	0.953115634	0.922470488
CTLA4	rs231726	0.966849098	0.932431744
TGFB1	rs8179181	0.979473716	0.940172394
IFNG	rs12369470	0.980717153	0.953292253
HLA-DQB1	rs7755224	0.992717878	0.96519939
	rs10484561	0.992717878	0.979146292
